# Assessment of sequence homology and immunologic cross-reactivity between tree shrew (*Tupaia belangeri*) and human IL-21

**DOI:** 10.1371/journal.pone.0176707

**Published:** 2017-05-03

**Authors:** Rong Ding, Hui Zhang, Lihong Zhang, Wenwen Zhao, Yongyin Li, Jianyong Yang, Yuanxu Zhang, Shiwu Ma

**Affiliations:** 1 Department of Infectious Diseases, Kunming General Hospital of Chengdu Military Region, Kunming, China; 2 State Key Laboratory of Organ Failure Research, Department of Infectious Diseases, Nanfang Hospital, Southern Medical University, Guangzhou, China; 3 Cell Biological Therapy Center, Kunming General Hospital of Chengdu Military Region, Kunming, China; 4 College of Life Science and Technology, Jinan University, Guangzhou, China; Fudan University, CHINA

## Abstract

Many studies have indicated that the expression of interleukin-21 (IL-21) is associated with the pathogenesis of certain liver diseases. However, in alternative animal models of liver diseases, it remains unknown whether the tree shrew could be utilized to analyze the relationship between IL-21 and liver diseases. Here, the phylogenetic tree, sequence alignment and protein structure model of tree shrew and human IL-21 were analyzed using bioinformatics software. A pEGFP-N3/tsIL-21 eukaryotic expression vector of tree shrew IL-21 (tsIL-21) was constructed, and IL-21 expression by the vector-transfected Huh7 cells was evaluated using the newly established quantitative real-time PCR and immunologic protocols for assessing human IL-21. The cytokine profiles were also evaluated in tree shrew spleen lymphocytes induced by recombinant human IL-21 or concanavalin A. It was found that the coding sequence (CDS) of tsIL-21 amplified from spleen lymphocytes belonged to the predicted sequence. The tsIL-21 was closely clustered with primate IL-21 rather than rodent IL-21, and it had an alignment of 83.33% with the human IL-21 nucleotide sequence and 69.93% with the amino acid sequence. The profiles of secondary structure, hydrophobicity and surface charge of tsIL-21 were also similar with those of human IL-21. The tsIL-21 expressed by the vector-transfected Huh7 cells could be identified by their different sources of antibodies against human IL-21, which were all dose-dependent. Recombinant human IL-21 could induce the change of the cytokine profiles of tree shrew spleen lymphocytes, which showed a higher expression of IL-10 and IFN-γ rather than IL-2, IL-4, IL-17, TNF-a and IL-21 during the five-day stimulation. These results indicate that tsIL-21 has a high degree of homology, structural similarity and immunological cross-reactivity with human IL-21 and also confirm the accuracy of this predicted tsIL-21CDS. The protocols utilized in this study will lead to the experimental feasibility of further IL-21-related studies in vivo.

## Introduction

Interleukin-21 (IL-21) is a type I cytokine that shares the common cytokine receptor γ chain with IL-2, IL-4, IL-7, IL-9, and IL-15[1]. It is produced by multiple CD4^+^ T cell subsets, including T helper 17 cells, follicular helper T cells, and natural killer T cells [1, 2]. IL-21 is thought of as a bridge between innate and adaptive immunity with immune-enhancing and immune-regulatory effects on B, T and natural killer cell responses [2, 3]. Recently, it was confirmed that IL-21 plays a pivotal role in controlling chronic viral infection [4]. In the field of hepatitis B virus (HBV)-related diseases, we found that HBV e antigen (HBeAg) seroconversion occurred in the antiviral therapy of chronic hepatitis B patients was closely related to IL-21[5], and the circulating CXCR5+CD4+ T cells benefit HBeAg seroconversion through IL-21 [6]. Studies from other groups, as well as ours, have also indicated that IL-21 is closely associated with the pathogenesis of liver fibrosis [7], liver failure [8] and hepatocellular carcinoma (HCC) [9]. As candidate agents, recombinant IL-21 and IL-21 receptor (IL-21R) antagonists have shown good efficacy and safety in clinical trials of carcinoma and autoimmune diseases [10–12]. In a recent macaque model study in vivo, recombinant IL-21 combined with antiretroviral therapy could reduce levels of simian immunodeficiency virus RNA and viral reservoirs [13]. Additionally, the in vivo study of IL-21-related liver diseases has been conducted in a transgenic mouse model, but the occurrence and development of human liver diseases, particularly hepatitis virus infection, could not be fully mimicked.

Tree shrews (*Tupaia belangeri*, family Tupaiidae) are small mammals and classified in the order Scandentia and the superorder Euarchonta. Compared to rats, mice, woodchucks, dogs and other laboratory animals, tree shrews have shown high degrees of similarity with humans in many aspects, such as metabolism, genomic and immunological characteristics [14]. Due to their small body size, high brain-to-body mass ratio, short reproductive cycle and life span and low cost of maintenance, the tree shrew has been proposed as an alternative experimental animal to primates in biomedical research, particularly in the research of certain liver diseases, such as chronic HBV and hepatitis C virus (HCV) infection, HCC [14–16]. Currently, it provides powerful support for the tree shrew as an ideal HBV infection animal model, when Na+/taurocholate cotransporting polypeptide (NTCP) was identified as the cellular receptor for HBV under the background of primary Tupaia hepatocytes [17]. In addition, the reported full gene sequence of the tree shrew provides a favorable background for genetics, molecular biology and immunology research [18]. However, few tree shrew-specific immunology research tools have been identified so far, which has limited further research on the immune mechanism.

Because of the overlap of the liver disease spectrum between the tree shrew and IL-21, it was speculated that promoting tree shrews as ideal animal models would be very valuable. Here, the genetic homology and immunologic cross-reactivity between tree shrew IL-21 (tsIL-21) and human IL-21 were evaluated, and several common immunological methods were established or evaluated for tree shrew research. The potential significance of this study aims to provide research tools as well as to explore an experimental basis for the further in vivo study of IL-21 in tree shrews.

## Material and methods

### Experimental animals and ethics statement

Chinese tree shrews were introduced from the experimental animal core facility of the Kunming Institute of Zoology, Chinese Academy of Sciences. Tree shrews were allowed free access to tap water and mixed provender and were housed in the cages in the experimental animal center of Kunming General Hospital of Chengdu Military Region. After they were lethally anesthetized by diethyl ether, we collected the spleens and blood. All animal experimental protocols were reviewed and approved by the Institutional Animal Care and Use Committee of Kunming Institute of Zoology, Chinese Academy of Sciences.

### Phylogenetic analysis

To evaluate the evolutionary conservation of the tsIL-21 gene, the coding sequences (CDS) of 18 species were aligned together and analyzed by DNAMAN 6.0 and MEGA 5.0 software. The involved species included tree shrew (*Tupaia chinensis*), human (*Homo sapiens*), gorilla (*Western gorilla*), chimpanzee (*Pan troglodytes*), monkey (*Macaca mulatta*), horse (*Equus caballus*), sheep (*Ovis aries*), cattle (*Bos taurus*), dog (*Canis lupus familiaris*), cat (*Felis catus*), guinea pig (*Cavia porcellus*), rabbit (*Oryctolagus cuniculus*), mouse (*Mus musculus*), rat (*Rattus norvegicus*), chicken (*Gallus*), mallard (*Anas platyrhynchos*), frog (*Xenopus tropicalis*) and zebrafish (*Danio rerio*). The three-dimensional structure models of tree shrew and human IL-21 proteins were deduced using Discovery Studio software.

### Cytokine primer design

All primers in this study (Table 1) were designed using an online primer design system (www.ncbi.nlm.nih.gov/tools/primer-blast/), specific assessments were acquired via the National Center for Biotechnology Information BLAST program (www.ncbi.nlm.nih.gov/BLAST) and synthesized by Sangon Biotech (Shanghai, China). Primers for tsIL-21 genes were designed based on the predicted sequence deposited in Genbank: tsIL-21 (XM_006153122.1). For full-length amplification, a pair of sequence-specific PCR primers of tsIL-21 were designed (IL-21-2, Table 1), which covered 25 base pairs of the head or the tail of tsIL-21 full length complementary DNA (cDNA) sequences, respectively. Another pair of tsIL-21 primers (IL-21-3, Table 1) were designed for plasmid construction, which included the restriction sites of HindIII or KpnI, respectively. For the quantitative real-time polymerase chain reaction (qRT-PCR) detection of tree shrew cytokine mRNA, the primers of IL-21 (IL-21-1, Table 1), IL-2, IFN-γ, IL-4, IL-10, IL-17, TNF-α and GAPDH were also designed.

**Table 1:** Primers used in the study.

Genes	Primer	Sequences 5’-3’	Application
GAPDH	GAPDH-F	GCTGGTGCTGAGTATGTTG	qRT-PCR
	GAPDH-R	AGTCTTCTGGGTGGCAGTGATG	qRT-PCR
IL-2	IL-2-F	GCACAAAATGCAACTCTTGTC	qRT-PCR
	IL-2-R	CCATTCAAAATCTTCTGTAAATCC	qRT-PCR
IFN-γ	IFN-γ-F	AGTATACAAGTTATACACTGG	qRT-PCR
	IFN-γ-R	GTCACTCTCCTCTGTCCAAT	qRT-PCR
TNF-α	TNF-α-F	ATCTGGAATCCCGAGTGACAAG	qRT-PCR
	TNF-α-R	CGTGAAGAGGACCTGGGAGTAG	qRT-PCR
IL-4	IL-4-F	GCAGACATCTTTGCCGCATC	qRT-PCR
	IL-4-R	ACCCATGGTGGCCATAGAAC	qRT-PCR
IL-10	IL-10-F	CAACTCAGCACTGCTATATTG	qRT-PCR
	IL-10-R	CTCAGCAACATGTTGTCCTG	qRT-PCR
IL-17	IL-17-F	TCCCCCAAACTGTGAACGTC	qRT-PCR
	IL-17-R	GCATCCACACAGCCCAAATG	qRT-PCR
IL-21	IL-21-1F	GGGACAGTGGCCCATAAATC	qRT-PCR
	IL-21-1R	GCCTTCTGAAAACATGAAAAAGC	qRT-PCR
	IL-21-2F	ATGAGATTCAGTCGTGGCAGCATGG	specific PCR amplification
	IL-21-2R	GGTGAGATCATCGGGAGCCTTCATA	specific PCR amplification
	IL-21-3F	CCCAAGCTTGGGATGAGATTCAGTCGTGGCAGCATGGAG ^a^	PCR for plasmid construction
	IL-21-3R	GGGGTACCCCTCAGGTGAGATCATCGGGAGCCTTCATATC	PCR for plasmid construction

^a^Restriction endonuclease sites introduced by PCR are underlined.

qRT-PCR, quantitative real-time PCR.

### Isolation and stimulation of tree shrew spleen lymphocytes

The spleen lymphocytes were obtained by density separation with mouse 1× Lymphocyte Separation Medium (DKW, Shenzhen, China) according to the manufacturer’s instructions. In the related experiments, cells were cultured with recombinant human IL-21 (ProSpec, Ness-Ziona, Israel) at a concentration of 50 ng/ml for 1 to 5 days, with concanavalin A (ConA; Sigma, St. Louis, MO, USA) at a concentration of 10 μg/ml for 1 day, or with medium only. The remaining cells and culture supernatant were harvested and stored at -80**°**C until further analysis by ELISA or qRT-PCR.

### tsIL-21 cDNA synthesis and amplification

Total RNA were extracted from ConA-induced spleen lymphocytes using Trizol reagent (TaKaRa, Shiga, Japan) and cDNA was synthesized using PrimeScript^™^ RT reagent Kit (TaKaRa, Shiga, Japan). For the amplification of tsIL-21 transcripts, the reaction was performed in a volume of 50 μl, consisting of 10 μl of 5× PrimeSTAR Buffer, 4 μl of dNTP Mixture (2.5 mM for each), 1 μl of each primer (IL-21-3, Table 1), 0.5 μl of Prime STAR HS DNA Polymerase (2.5 U/μl), 2 μl of cDNA. PCR was performed as follows: pre-denaturation for 5 min at 98**°**C, denaturation for 10 s at 98**°**C, annealing for 15 s at 58**°**C, and extension for 30 s at 72**°**C; a total of 30 cycles was used, followed by extension for 10 min at 72**°**C. Purified PCR products were sequenced by Sangon Biotech (Shanghai, China).

### Construction of eukaryotic expression vectors pEGFP-N3/tsIL-21

The eukaryotic expression vectors pEGFP-N3 (Clontech, CA, USA) and tsIL-21 transcripts were digested by Hind III and Kpn I (NEB, Ipswich, MA, USA) at 37**°**C for 2 h. The tsIL-21 fragments were cloned into pEGFP-N3 vectors by T4 DNA ligase (TaKaRa, Japan). The pEGFP-N3/tsIL-21 plasmids were transformed into *Escherichia coli* DH5α competent cells (TIANGEN, Beijing, China). The plasmid was extracted using the TIANprep Mini Plasmid Kit (TIANGEN, Beijing, China) and identified by specific PCR amplification with the primer of IL-21-2F and IL-21-2R (Table 1). The PCR products were also confirmed by sequencing.

### Cell transfection and immunofluorescence

Huh7 cells were cultured in DMEM medium (Gibco, GrandIsland, NY, USA) containing 100 U/ml penicillin, 100 mg/ml streptomycin and 10% FBS (Gibco, GrandIsland, NY,USA). When the cultured cells reached 70%-90% confluence, they were sub-cultured at a ratio of 1:2 using trypsin. As the experimental group (EG), the pEGFP-N3/tsIL-21 plasmid was transfected into Huh7 cells using Lipofectamine 3000 (Invitrogen, Carlsbad, CA, USA) with Opti-MEM (Gibco, GrandIsland, NY, USA); meanwhile, the empty plasmid control group (CG) and the blank group (BG) were set up by transfecting pEGFP-N3 plasmid or the medium, respectively. Transfection conditions were optimized by varying the cell density and the concentration of plasmid DNAs for pEGFP-N3/tsIL-21 and Lipofectamine 3000 (Invitrogen, Carlsbad, CA, USA) according to the manufacturer’s instructions to obtain the highest transfection efficiency and lowest cytotoxicity. The transfection efficiency and expression were assessed under a fluorescence microscope at 6, 24, 48, and 72 h, respectively.

### Quantitative real-time PCR (qRT-PCR)

The qRT-PCR was performed using an UltraSYBR mixture (with ROX I, CWBIO, Guangzhou, China) and cytokine-specific primers (Table 1). The reaction was performed in volume of 20 μl, consisting of 10 μl of 2× UltraSYBR mixture, 0.4 μl of Primer F/R (0.2 μM for each), 1 μl of cDNA, and RNase-free water up to 20 μl. At the same time, the negative control group without cDNA was established. The qRT-PCR was performed as follows: pre-denaturation for 10 min at 95**°**C, denaturation for 15 s at 95**°**C, annealing and extension for 1 min at 60**°**C; a total of 40 cycles was used following dissolution curve analysis: 15 s at 95**°**C, 1 min at 60**°**C, 15 s at 95**°**C and 15 s at 60**°**C. The 2^−ΔΔCT^ method was used for processing [19]. The average values of five independent experimental data sets were taken for each sample.

### Intracellular cytokine staining (ICS)

The four groups of Huh7 cell mixture suspensions were set up according to Huh7 cells transfected with different ratios of the pEGFP-N3 plasmid to the pEGFP-N3/tsIL-21 plasmid (CG:EG = 3:0, 2:1, 1:2, 0:3, respectively). A minimum of 10^6^ cells were collected, and the intracellular cytokine staining was performed using Fix & Perm (Invitrogen, Carlsbad, CA, USA), mouse-anti-human IL-21-PE and its isotype (BioLegend, San Diego, CA, USA). The data were analyzed by FACSDiva (BD Bioscience, San Jose, CA) and FlowJo software (Tree Star Inc., Ashland, OR). A similar procedure was conducted in the ICS assay of tree shrew spleen lymphocytes.

### Enzyme-linked immunosorbent assay (ELISA)

A commercial human IL-21 ELISA kit (eBioscience, San Diego, CA, USA) was used to measure IL-21 concentrations within the tree shrew serum and the culture supernatant of tree shrew spleen lymphocytes, as well as the above four types of cell mixture suspensions after five rounds of freezing and thawing. Quantification was performed using a standard curve generated using a known concentration of recombinant human IL-21, and the lower detection limit of the kit was 20 pg/ml.

### Western blotting (WB)

The proteins expressed by the transfected Huh7 cells were extracted using Mammalian Protein Extraction Kit (CWBIO, Guangzhou, China). The samples were electrophoresed in SDS-PAGE (12%) and transferred to a polyvinylidene fluoride membrane under 90 V constant voltage for 1 h. The membrane was stained with rabbit-anti-human IL-21 antibody (Abcam, Cambridge, MA, USA) and then secondary HRP-conjugated goat-anti-rabbit IgG (Abcam, Cambridge, MA, USA) and colored substrate (BCIP/NBT).

### Statistical analysis

The results were statistically analyzed using SPSS 17.0 software. Experimental data were expressed as medians (range). Wilcoxon’s signed-rank test was used when two groups were compared, and the Kruskal-Wallis H test was used when more than two groups were compared. All statistical analyses were based on two-tailed hypothesis tests with a significance level of *p*<0.05.

## Results

### Homology analysis of tsIL-21

**Fig 1:**
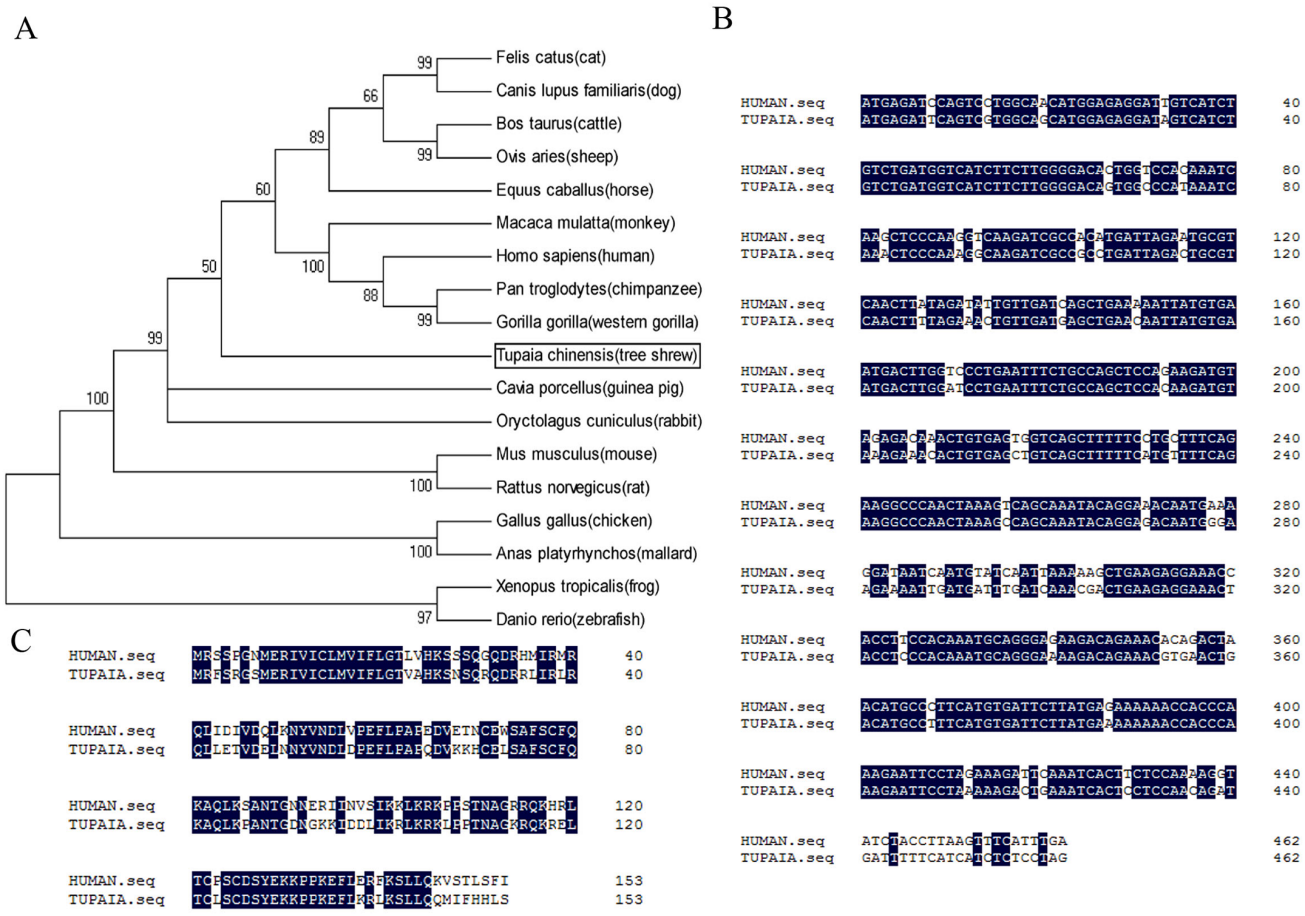
Homology analysis of the IL-21 gene. (A) Phylogenetic tree of the IL-21 gene based on the amino acid sequences among eighteen species. Alignment of tree shrew and human IL-21 coding sequences (B) and amino acid sequences (C).

**Fig 2:**
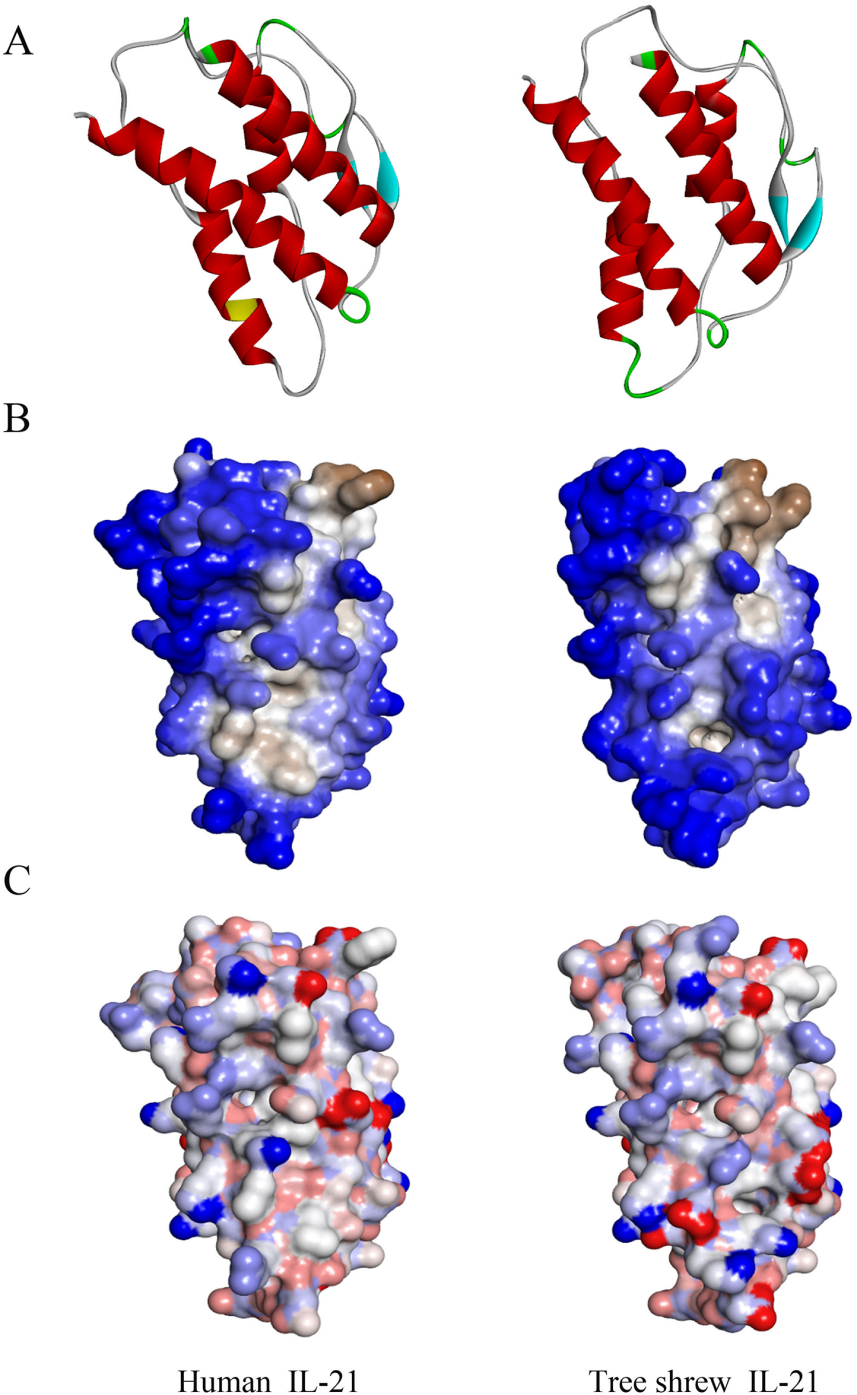
Predicted three-dimensional structures of the tree shrew and human IL-21 protein. (A) Secondary structure of tree shrew IL-21 (right) compared with human IL-21 (left). Red represents α helices, cyan represents β sheets, green represents β turns, white represents random coils, and yellow represents N-glycosylation sites. (B) Hydrophobicity of tree shrew IL-21 (right) compared with human IL-21 (left). Blue represents hydrophilicity, brown represents hydrophobicity, white represents transition. (C) Surface charge of tree shrew IL-21 (right) compared with human IL-21 (left). Blue represents negative charge, red represents positive charge, white represents no electrical charge.

The phylogenetic tree showed that the tsIL-21 transcripts was first clustered with human, monkey, sheep and cattle and secondly with mouse and rat (Fig 1A), which demonstrated that human IL-21 had a closer affinity to tsIL-21 than the rodents’ IL-21. Furthermore, to explore the evolutionary conservation of tsIL-21, we compared the IL-21 CDS and amino acid sequences between humans and tree shrews, and the results showed that both tree shrew and human IL-21 contained a 462-nucleotide (nt) CDS (Fig 1B), which could represent a 153-amino-acid (aa)-length protein (Fig 1C). Although it was not completely consistent between the tsIL21 (Genbank. XM_006153122) and human IL-21 CDS (Genbank.NM_001207006), the alignment was 83.33% (385/462) with the nucleotide sequence and 69.93% (107/153) with the amino acid sequence. In addition, the three-dimensional structure models of tree shrew and human IL-21 proteins were constructed (Fig 2A). The secondary structure of the tsIL-21 protein was similar to that of the human IL-21 protein. The number of alpha helices was approximately same in tree shrew and human IL-21, as well as the position of the alpha helix and beta folding. However, there was a N-glycosylation site in human IL-21, which was not found in tsIL-21 (Fig 2A). Then, we analyzed and predicted the difference in hydrophobicity and surface charge distribution between tree shrew and human IL-21. The results showed that both tree shrew and human IL-21 protein molecules were hydrophobic, but only in individual regions, tsIL-21 was partially hydrophilic, whereas human IL-21 was partially hydrophobic (Fig 2B). The surface charges on tree shrew and human IL-21 were also similar. However, within the 60th~70th area of the amino acid sequence, the human IL-21 surface charge was less than that of tsIL-21 (Fig 2C). These results suggested that IL-21 exhibited good gene homology and structural similarity between tree shrews and humans.

### Construction, transfection and expression of the pEGFP-N3/tsIL-21 plasmid

To detect whether there was cross-reactivity between tree shrew and human IL-21, a eukaryotic expression vector was designed (S1 Fig). The IL-21 full-length cDNA sequence was successfully amplified from ConA-induced tree shrew spleen lymphocytes and matched with the published IL-21 cDNA sequence on PubMed. The constructed pEGFP-N3/tsIL-21 was confirmed by double enzyme digestion (S1 Fig), specific PCR amplification (S1 Fig) and sequencing. During the observation of transfection effects, the green fluorescent protein (GFP) was shown in the EG group as well as in the CG group, but not in the BG group. However, the green fluorescence was weaker in the EG group than in the CG group at the time points of 6 h, 24 h, 48 h and 72 h (S1 Fig). Thus, to demonstrate whether the transfection of the pEGFP-N3/tsIL-21 plasmid affected the morphology and proliferation rates of Huh7 cells, further observation was carried out using an ordinary light microscope. It was shown that the Huh7 cells in the EG, CG and BG groups all grew well, which meant that a dense monolayer was formed at 48 h without differences in the morphology and proliferation rate among the three groups (S1 Fig).

To test the transfected tsIL-21 gene expression, the RNAs were extracted from three groups of Huh7 cells 48 h after transfection. Through PCR amplification with tree shrew IL-21-specific primers, a 498-bp specific fragment was shown in the EG group, but not in the CG and BG group. Furthermore, we also detected the relative mRNA expression of tsIL-21 in Huh7 cells by qRT-PCR, and the results illustrated that the expression level of tsIL-21 mRNA in Huh7 cells was high in the EG group, but no expression was found in the CG and BG groups (S1 Fig).

### Detection of recombinant tree shrew IL-21 expression using antibodies against human IL-21

**Fig 3:**
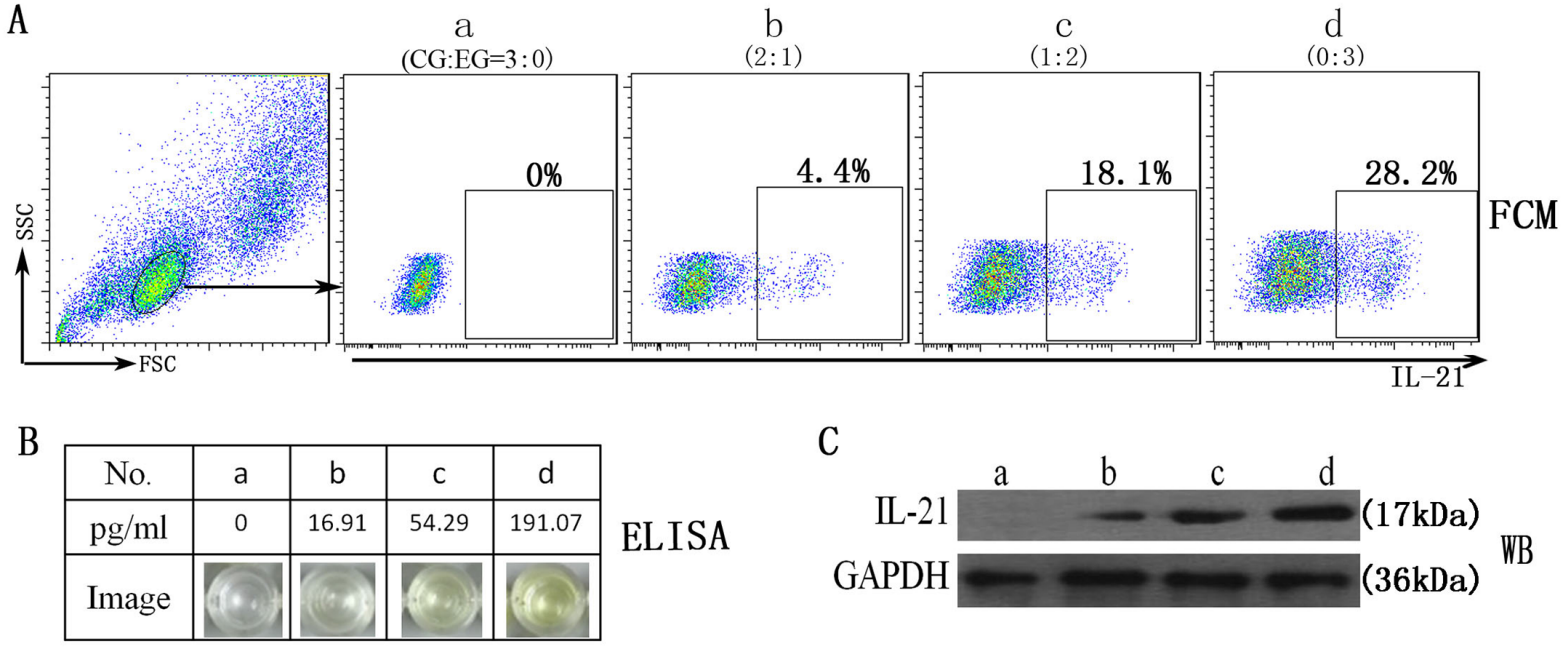
The reactivity of anti-human IL-21 antibody to tree shrew IL-21 expressed by transfected Huh7 cells. (A) FCM represents flow cytometry staining; (B) ELISA represents enzyme-linked immunosorbent assay; (C) WB represents western blotting. CG represents the control group with pEGFP-N3 plasmid transfection; EG represents the experimental group with pEGFP-N3/tsIL-21 plasmid transfection.

Due to the lack of availability of a tsIL-21-specific antibody, it was considered that the IL-21 expression of the transfected Huh7 cells was determined by commercial reagents against human IL-21, and the cross-reactivity of IL-21 was also evaluated. The four groups of cell mixture suspensions (a, b, c and d) were set up according to the ratio of the pEGFP-N3 plasmid to the pEGFP-N3/tsIL-21 plasmid-transfected Huh7 cells (CG:EG = 3:0, 2:1, 1:2, 0:3, respectively). The IL-21 expression of four groups of Huh7 cells was determined by the strategies of flow cytometry staining (Fig 3A), enzyme-linked immunosorbent assay (Fig 3B) and western blotting (Fig 3C) using three types of commercial reagents. With the increase of pEGFP-N3/tsIL-21 plasmid concentration, group D showed more expression of tree shrew IL-21 than the other three groups. In addition, the dose-dependent tendency between the concentration of pEGFP-N3/tsIL-21 plasmids and the expression of tsIL-21 was shown in all three types of immunology assays. Therefore, these results suggested that the antibody against human IL-21 could have good cross-reactivity with tsIL-21.

### Detection of IL-21 expression in tree shrew samples

**Fig 4:**
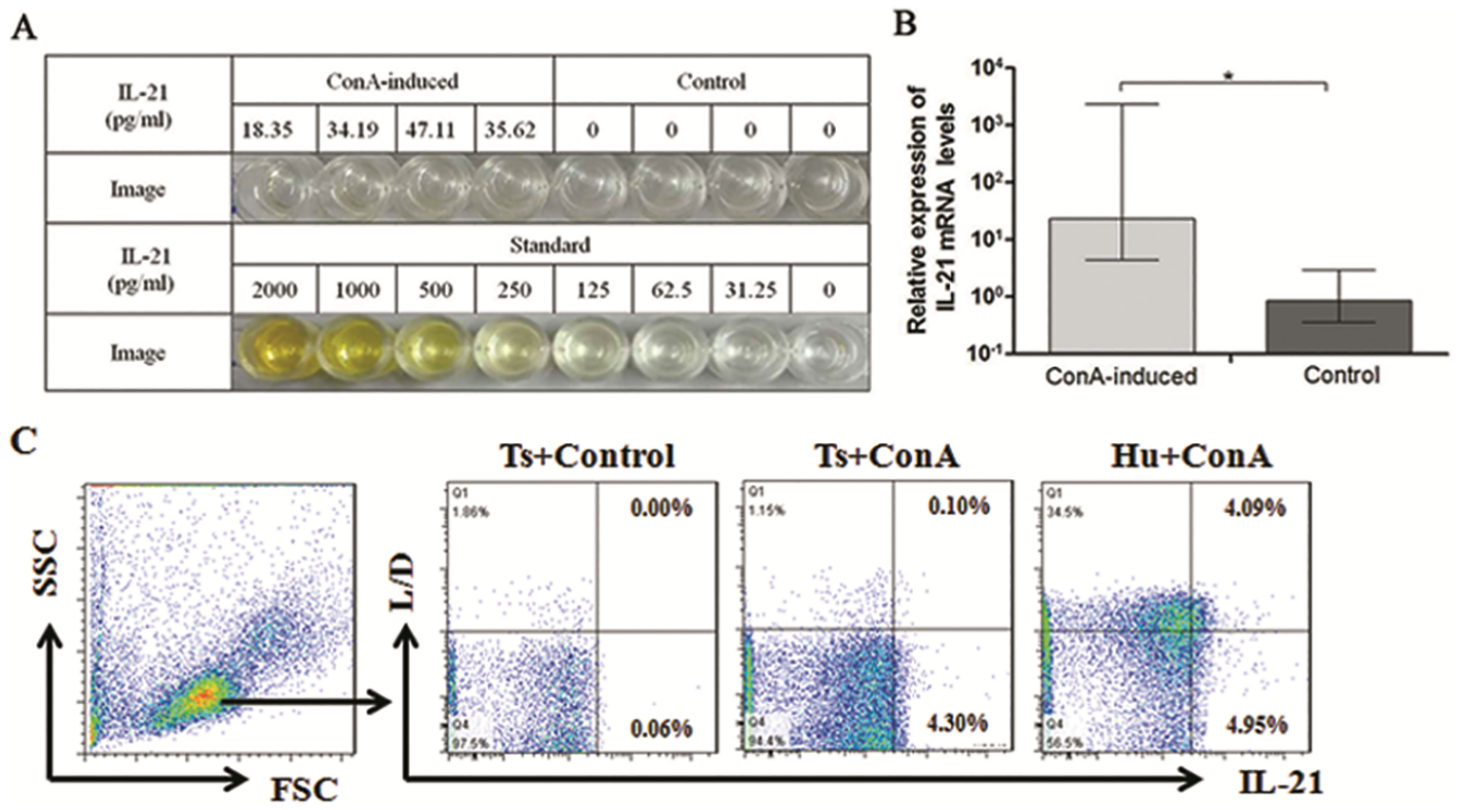
The IL-21 expression in tree shrew samples. (A) Concentrations of IL-21 in supernatant from tree shrew spleen lymphocytes induced with ConA by ELISA. (B) Quantification of IL-21 mRNA expression in tree shrew spleen lymphocytes induced by ConA. *represents a significant difference compared to stimulation with ConA and the unstimulated negative control (*P*<0.05). (C) The IL-21 expression of tree shrews spleen lymphocytes and human peripheral blood mononuclear cells were detected by flow cytometry staining.

To further validate IL-21’s cross-reactivity, the expression of IL-21 in tree shrew samples was detected using a human IL-21 ELISA kit. The concentrations of IL-21 were from 18.35 pg/ml to 47.11 pg/ml in the culture supernatants of ConA-induced spleen lymphocytes, whereas there was no positive reaction in the supernatants of the controls group without ConA (Fig 4A). Meanwhile, IL-21 mRNA quantifications were also determined by qRT-PCR through the corresponding spleen lymphocytes, and the results also demonstrated that the expression of IL-21 mRNA in the ConA-induced group was significantly higher than that in the control group (Fig 4B). In addition, the serum concentrations of tsIL-21 in 9 adult healthy tree shrews, were also determined using a human IL-21 ELISA kit, only one serum sample’s IL-21 level more than the lower detection limit of the kit, and the results indicated that the serum concentrations of tsIL-21 were very low in adult healthy tree shrews, ranging from 0 to 30.47 pg/ml. However, after ConA stimulation in vitro, both tree shrew spleen lymphocytes and human peripheral blood mononuclear cells could significantly expressed IL-21 (Fig 4C).

### Effects of recombinant human IL-21(rhIL-21) on the cytokine profiles of tree shrew spleen lymphocytes

**Fig 5:**
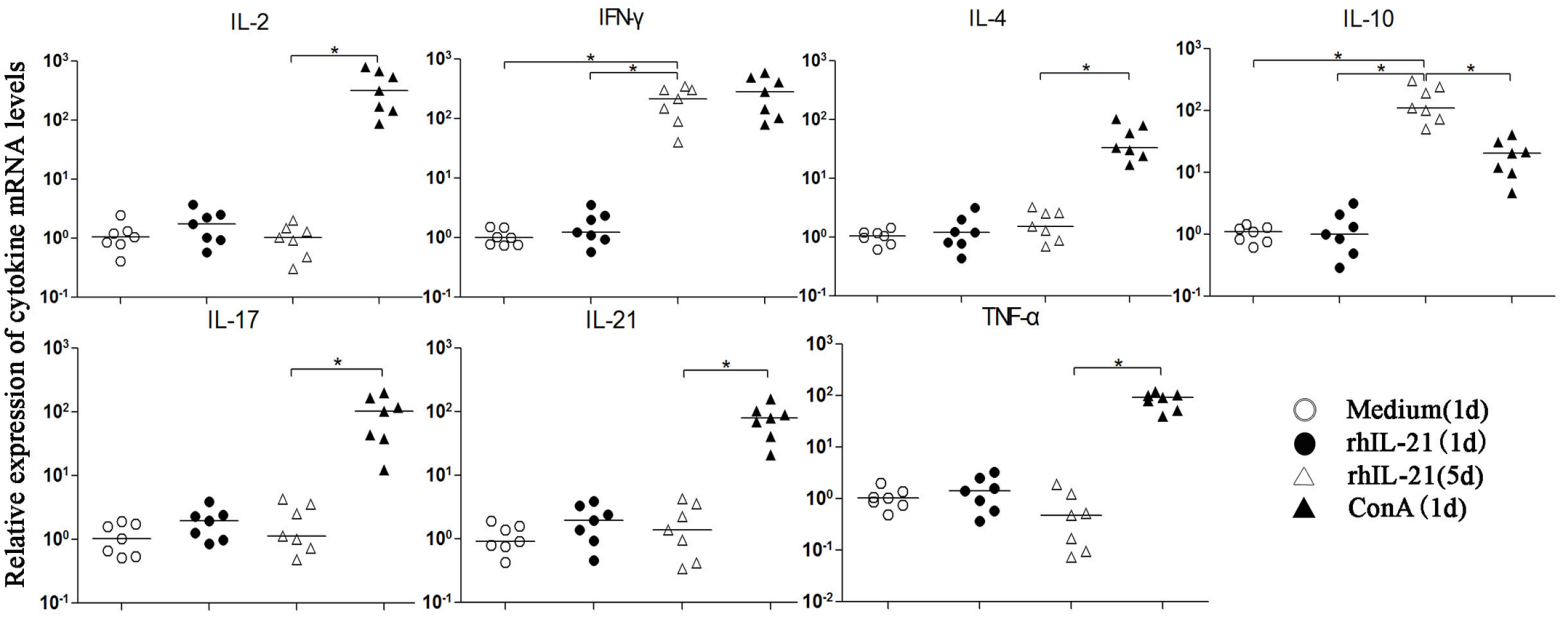
Effects of recombinant human IL-21 on the cytokine profiles of tree shrew spleen lymphocytes. *represents a statistically significant difference between two groups (*P*<0.05).

Furthermore, to explore the effects of rhIL-21 on the tree shrew cytokine profile, the spleen lymphocytes of tree shrews were induced by rhIL-21, ConA and medium, respectively. The mRNA expressions of IL-2, IFN-γ, IL-4, IL-10, IL-17, TNF-α and IL-21 were determined using the newly established qRT-PCR method (Fig 5). The rhIL-21 could induce changes in the cytokine spectrum of tree shrew spleen lymphocytes, which showed higher expression levels of IL-10 and IFN-γ rather than IL-2, IL-4, IL-17, TNF-a and IL-21 during a five-day stimulation. Furthermore, the expressions of all seven cytokines were significantly up-regulated upon stimulation with ConA compared with the medium control. However, the expression of IL-10 was significantly higher in rhIL-21 for five days than that in ConA for one day (*P*<0.05), and the expression of INF-γ showed the opposite tendency (*P*<0.05).

## Discussion

In molecular and cellular profiles, biological phenomena usually follow similar or identical basic rules. Therefore, when studying the molecular and cellular mechanisms of important biological problems and diseases, lower animals obviously have unique advantages. Although these molecular and cellular mechanisms are similar to those of humans on the unitary level, higher animals must be studied as well. Genome analysis has indicated that tree shrews have high degrees of homology with humans in immunology [18] and liver protein expression profiles [20], and the evolutionary distance to humans is also closer for tree shrews than for rodents. In this study, we completed the phylogenetic analysis and the sequence comparison of the IL-21 gene and analyzed the similarities in the secondary structure, hydrophobicity and surface charge of the predicted IL-21 protein between tree shrews and humans. We also found these similarities in IL-21 receptor (IL-21R) between tree shrew and human (S2–S4 Figs), and the alignment was 80.49% with the nucleotide sequence and 73.48% with the amino acid sequence. Particularly, the predicted three-dimensional structure of tsIL-21R can well overlap with that of human IL-21R in very low root-mean-square error (4.414Å). The results further confirm the high degree of homology in the immunology between humans and tree shrews.

Currently, in addition to humans and chimpanzees, tree shrews are the only animals that can be infected by both HBV and HCV. As is known, chimpanzees are difficult to develop into an ideal animal model because of the scarcity of resources coupled with economic and ethical problems. Hepatitis virus genes can be transduced into transgenic mice, but the process of virus infection cannot be simulated. Although the human hepatocyte chimeric mouse can be infected by HBV and HCV, it cannot be used for the study of the inflammatory and immunology reactions [21]. Except for HBV and HCV, tree shrews are also potential infection models for other viruses [22]. Therefore, available detection tools are necessary for choosing tree shrews as experimental animal models. Especially in immunology research, it is important to develop enough reagents to detect the expression levels of genes and proteins. As a new animal model, the genome of the tree shrew has just been revealed [18]; thus, related immunology research tools are urgently needed. Recently, the literature has described some tree shrew cytokines and molecules, such as CXCR4, IL-7, IL-2 and GAPDH [23–26]; however, the cross-reactivity phenomenon was not clearly revealed until now. Here, our study on the homology and cross-reactivity of tsIL-21 will be an important supplement to immunology research on the tree shrew.

At present, the available reference sequence of tsIL-21 remains incomplete. In this study, the referred tsIL-21 CDS was based on a predicted sequence in Genbank (XM_006153122.1). Therefore, the accuracy of this predicted sequence should firstly be tested using experiments. In this study, the tsIL-21 CDS transcript was amplified from healthy tree shrew spleen lymphocytes, and the sequencing result was strictly in accordance with the predicted tsIL-21 sequence. Additionally, the tsIL-21 expressed by the pEGFP-N3/tsIL-21 transfected Huh7 cells and the ConA-induced spleen lymphocytes showed good cross-reactivity with anti-human IL-21 antibodies. These results illustrated that the predicted tsIL-21 sequence was accurate, which laid a solid foundation for the further application of this sequence and the further study of tsIL-21. Certainly, the variations in the whole tsIL-21 gene remain unknown because the non-coding sequence and regulatory sequence of the tsIL-21 gene were not involved here.

This study indicated not only that there was cross-reactivity between tree shrew and human IL-21 but also that this type of cross-reactivity exhibited a dose-dependent effect. This suggested that the human IL-21-related reagents could be alternatives to the qualitative and quantitative analysis of tsIL-21 when there is no available anti-tsIL-21 antibody. In addition, a set of qRT-PCR protocols was also introduced for the detection of tsIL-21 related cytokines (Th1/Th2/Th17). Therefore, these methods should meet the basic demand for tree shrew immunology research. In our study, IL-21 expression was very low in both the serum and spleen lymphocytes of healthy tree shrews by qRT-PCR for tree shrews and ELISA for humans, which were similar to the levels of healthy people in previous investigations [5]. The cytokine expression profile could be changed when tree shrew spleen lymphocytes were induced by ConA, in which tree shrew IL-21 was up-regulated. Interestingly, ConA could induce liver injury in the mouse model, which was associated with T cell activation and autophagy [27, 28]; therefore, it might be meaningful to evaluate the relationship between these cytokines and ConA-induced liver injury in a tree shrew model.

The IL-21 protein produced by the pEGFP-N3/tsIL-21-transfected Huh7 cells was different from IL-21 produced by ConA-induced tree shrew spleen lymphocytes because the former was the fusion protein but not the secreted protein. Therefore, to expose the IL-21 protein epitope to the anti-IL-21 antibody, the transfected Huh7 cells must be treated through lysis or perforation before IL-21 cross-reactivity experiments. The eukaryotic expression vector must be further modified to acquire purified tsIL-21. In that case, recombinant tsIL-21 can be utilized to treat human lymphocytes, and the cross-reactivity of tree shrew and human IL-21 can be further analyzed.

In conclusion, our study indicated that tsIL-21 has a high degree of homology, structural similarity and immunological cross-reactivity with human IL-21. Our results also confirm the accuracy of the predicted tree shrew IL-21 CDS. The protocols utilized in this study, which directly detected the tree shrew IL-21 gene by qRT-PCR and indirectly detected the tsIL-21 protein by anti-human IL-21 reagents, will support the experimental feasibility of further IL-21-related in vivo studies.

## Supporting information

S1 Fig The design, identification and expression of eukaryotic expression vectors pEGFP-N3/tsIL-21. (A) Map of plasmid pEGFP-N3/tsIL-21 construction. The red fragment indicated the insertion of a tsIL-21 gene sequence. (B) The products of pEGFP-N3/tsIL-21 after HindIII/KpnI enzyme digestion. Lane M represents the DNA marker; lanes 1 and 2 represent the digested pEGFP-N3/tsIL-21 plasmid. (C) The PCR amplification products of the pEGFP-N3/tsIL-21 plasmid. Lane M represents the DNA marker; lanes 1 and 2 represent the fragments of the tsIL-21 gene. (D) Observation of the transfected Huh7 cells by fluorescence microscopy over time. BG represents the blank group without transfection; EG represents the experimental group with pEGFP-N3/tsIL-21 plasmid transfection; CG represents the control group with pEGFP-N3 plasmid transfection. (E) Effects of pEGFP-N3/tsIL-21 plasmid transfection on the activity of Huh7 cells (×100). (F) Tree shrew IL-21 mRNA expression in transfected Huh7 cells. *represented *P*<0.05.
(TIF)

S2 Fig Alignment of tree shrew and human IL-21R coding sequences. (TIF)

S3 Fig Alignment of tree shrew and human IL-21R amino acid sequences. (TIF)

S4 Fig Predicted three-dimensional structures of the tree shrew and human IL-21R protein. (A) Secondary structure of tree shrew IL-21R (right) compared with human IL-21R (left). Red represents α helices, cyan represents β sheets, green represents β turns, white represents random coils, and yellow represents N-glycosylation sites. (B) Hydrophobicity of tree shrew IL-21R (right) compared with human IL-21R (left). Blue represents hydrophilicity, brown represents hydrophobicity, white represents transition. (C) Surface charge of tree shrew IL-21R (right) compared with human IL-21R (left). Blue represents negative charge, red represents positive charge, white represents no electrical charge.
(TIF)

## References

[1] Parrish-Novak J, Dillon SR, Nelson A, Hammond A, Sprecher C, Gross JA, et al Interleukin 21 and its receptor are involved in NK cell expansion and regulation of lymphocyte function. Nature. 2000; 408: 57–63. 10.1038/35040504 11081504

[2] Spolski R, Leonard WJ. Interleukin-21: basic biology and implications for cancer and autoimmunity. Annu Rev Immunol. 2008; 26: 57–79. 10.1146/annurev.immunol.26.021607.090316 17953510

[3] Monteleone G, Pallone F, Macdonald TT. Interleukin-21 as a new therapeutic target for immune-mediated diseases. Trends Pharmacol Sci. 2009;30: 441–447. 10.1016/j.tips.2009.05.006 19616319

[4] Johnson LDS, Jameson SC. A chronic need for IL-21. Science. 2009;324: 1525–1526. 10.1126/science.1176487 19541985

[5] Ma SW, Huang X, Li YY, Tang LB, Sun XF, Jiang XT, et al High Serum IL-21 Levels Within Initial 12 Weeks of Treatment Predict HBeAg Seroconversion During Antiviral Therapy in Chronic Hepatitis B. J Hepatol.2012;56: 775–781. 10.1016/j.jhep.2011.10.020 22173154

[6] Li YY, Ma SW, Tang LB, Li Y, Wang W, Huang X, et al Circulating CXCR5+CD4+T cells Benefit HbeAg Seroconversion Through IL-21 in Patients with Chronic HBV Infection. Hepatology, 2013;58:1277–1286. 10.1002/hep.26489 23703545

[7] Feng G, Zhang JY, Zeng QL, Yu X, Zhang Z, Lv S, et al Interleukin-21 mediates hepatitis B virus-associated liver cirrhosis by activating hepatic stellate cells. Hepatol Res. 2014; 44: 198–205. 10.1111/hepr.12215 23905760

[8] Hu XX, Ma SW, Huang X, Jiang XT, Zhu XL, Gao HB, et al Interleukin-21 Was Upregulated in Hepatitis B Related Acute-on-Chronic Liver Failure and Associated with Severity of Disease. J Viral Hepat. 2011;18: 458–467. 10.1111/j.1365-2893.2011.01475.x 21692955

[9] Pang YL, Zhang HG, Peng JR, Pang XW, Yu S, Xing Q, et al The immunosuppressive tumor microenvironment in hepatocellular carcinoma. Cancer Immunol Immunother. 2009;58: 877–886. 10.1007/s00262-008-0603-5 18941744 PMC11030619

[10] Stolfi C, Pallone F, Macdonald TT, Monteleone G. Interleukin-21 in cancer immunotherapy: Friend or foe? Oncoimmunology. 2012;1: 351–354. 10.4161/onci.19122 22737612 PMC3382872

[11] Hua F, Comer GM, Stockert L, Jin B, Nowak J, Pleasic-Williams S, et al Anti-IL21 receptor monoclonal antibody (ATR-107): Safety, pharmacokinetics, and pharmacodynamic evaluation in healthy volunteers: A phase I, first-in-human study. J Clin Pharmacol. 2014;54: 14–22. 10.1002/jcph.158 23913720

[12] Zanin-Zhorov A, Weiss JM, Nyuydzefe MS, Chen W, Scher JU, Mo R et al Selective oral ROCK2 inhibitor down-regulates IL-21 and IL-17 secretion in human T cells via STAT3-dependent mechanism. Proc Natl Acad Sci U S A. 2014;111: 16814–16819. 10.1073/pnas.1414189111 25385601 PMC4250132

[13] Micci L, Ryan ES, Fromentin R, Bosinger SE, Harper JL, He T, et al Interleukin-21 combined with ART reduces inflammation and viral reservoir in SIV-infected macaques. J Clin Invest. 2015;125: 4497–4513. 10.1172/JCI81400 26551680 PMC4665780

[14] Tsukiyama-Kohara K, Kohara M. Tupaia belangeri as an experimental animal model for viral infection. Exp Anim.2014;63: 367–374. 10.1538/expanim.63.367 25048261 PMC4244285

[15] Yang EB, Cao J, Su JJ, Chow P. The tree shrews: useful animal models for the viral hepatitis and hepatocellular carcinoma. Hepatogastroenterology. 2005; 52: 613–616. 15816489

[16] Yang ZF, Zhao J, Zhu YT, Wang YT, Liu R, Zhao SS, et al The tree shrew provides a useful alternative model for the study of influenza H1N1 virus. Virol J. 2013;10: 1–9. 23575279 10.1186/1743-422X-10-111 PMC3639867

[17] Yan H, Zhong G, Xu G, He W, Jing Z, Gao Z, et al Correction: Sodium taurocholate cotransporting polypeptide is a functional receptor for human hepatitis B and D virus. Elife. 2014; 3: e05570. 10.7554/eLife.00049 25409679

[18] Fan Y, Huang ZY, Cao CC, Chen CS, Chen YX, Fan DD, et al Genome of the Chinese tree shrew. Nat Commun. 2013; 4:1426 10.1038/ncomms2416 23385571

[19] Livak KJ, Schmittgen TD. Analysis of relative gene expression data using real-time quantitative PCR and the 2− ΔΔCT method. Methods. 2001; 25: 402–408. 10.1006/meth.2001.1262 11846609

[20] Li RX, Xu W, Wang Z, Liang B, Wu JR, Zeng R. Proteomic characteristics of the liver and skeletal muscle in the Chinese tree shrew (Tupaia belangeri chinensis). Protein Cell. 2012; 3(9): 691–700. 10.1007/s13238-012-2039-0 22886497 PMC4875369

[21] Chayama K, Hayes CN, Hiraga N, Abe H, Tsuge M, Imamura M. Animal model for study of human hepatitis viruses. J Gastroenterol Hepatol. 2011 1; 26(1):13–18. 10.1111/j.1440-1746.2010.06470.x 21175788

[22] Wang XX, Li JX, Wang WG, Sun XM, He CY, Dai JJ. Preliminary investigation of viruses to the wild tree shrews (Tupaia belangeri chinese). Zool Res. 2011; 32(1): 66–69. 10.3724/SP.J.1141.2011.01066 21341387

[23] Zheng Y, Wang Q, Yun C, Wang Y, Smith WW, et al Identification of glyceraldehyde 3-phosphate dehydrogenase sequence and expression profiles in tree shrew (Tupaia belangeri). PLoS ONE. 2014; 9(6): e98552 10.1371/journal.pone.0098552 24887411 PMC4041755

[24] Chen G, Wang W, Meng S, Zhang L, Wang W, et al CXC chemokine CXCL12 and its receptor CXCR4 in tree shrews (Tupaiabelangeri): Structure, Expression and Function. PLoS ONE. 2014; 9(5): e98231 10.1371/journal.pone.0098231 24858548 PMC4032326

[25] Yu D, Xu L, Liu XH, Fan Y, Lu LB, Yao YG. Diverse interleukin-7 mRNA transcripts in Chinese tree shrew (Tupaia belangeri chinensis). PLoS ONE. 2014; 9(6):e99859 10.1371/journal.pone.0099859 24945249 PMC4063794

[26] Huang XY, Li ML, Xu J, Gao YD, Wang WG, Yin AG, et al Analysis of the molecular characteristics and cloning of full-length coding sequence of Interleukin-2 in tree shrews. Zool Res. 2013; 4 34(2):121−126 10.3724/SP.J.1141.2013.02121 23572362

[27] Yang MC, Chang CP, Lei HY. Endothelial cells are damaged by autophagic induction before hepatocytes in Con A-induced acute hepatitis. Int Immunol. 2010 8;22(8):661–70. Epub 2010 Jun 13. 10.1093/intimm/dxq050 20547544

[28] Li J, Chen K, Li S, Liu T, Wang F, Xia Y, et al Pretreatment with fucoidan from fucus vesiculosus protected against ConA-induced acute liver injury by inhibiting both intrinsic and extrinsic apoptosis. PLoS ONE. 2016 4 1;11(4):e0152570 eCollection 2016. 10.1371/journal.pone.0152570 27035150 PMC4818100

