# Supplementary figures and images for: Assessment of sequence homology and immunologic cross-reactivity between tree shrew (Tupaia belangeri) and human IL-21
Source: PLoS One. 2017 May 3;12(5):e0176707. doi: 10.1371/journal.pone.0176707 (PMC5415133; doi:10.1371/journal.pone.0176707)

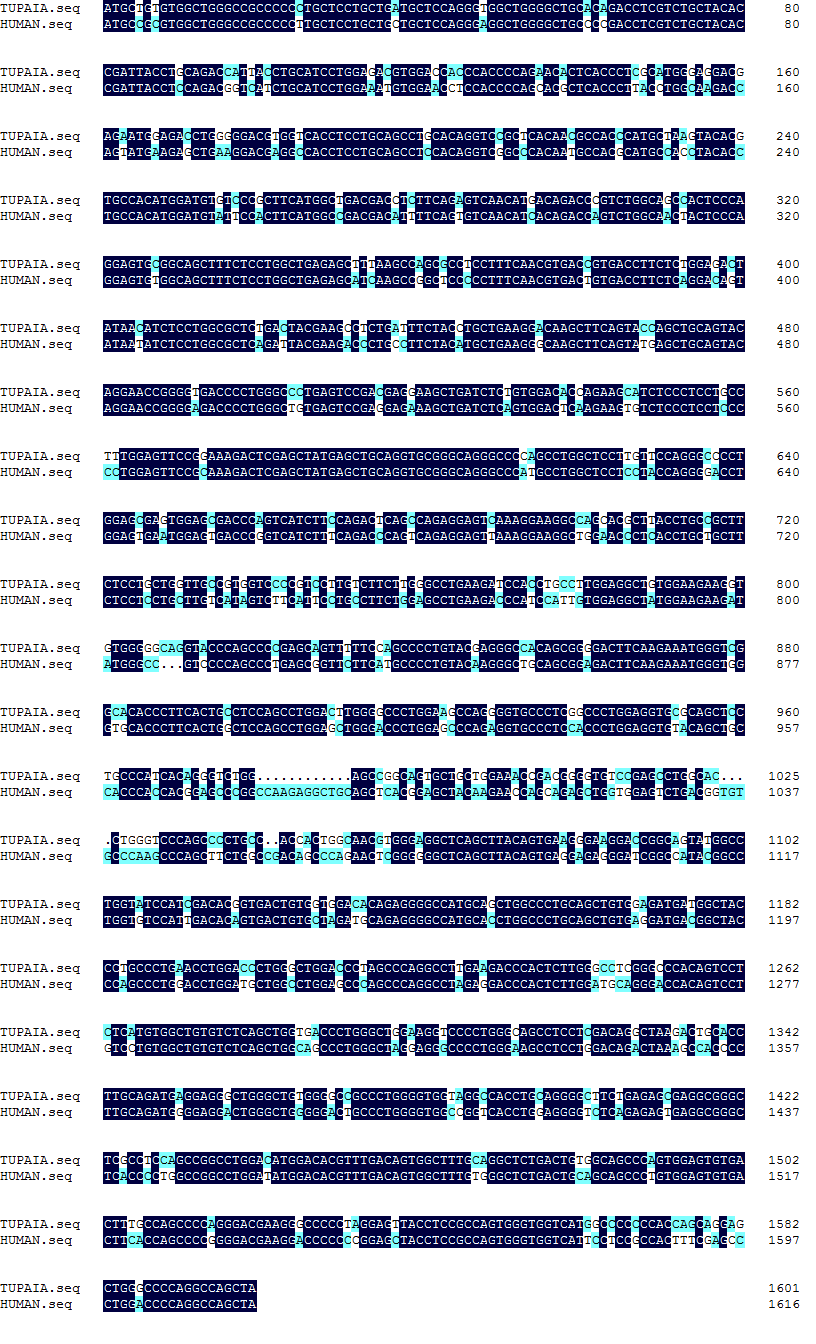

Supplement: S2 Fig — (TIF) [file pone.0176707.s002.tif]

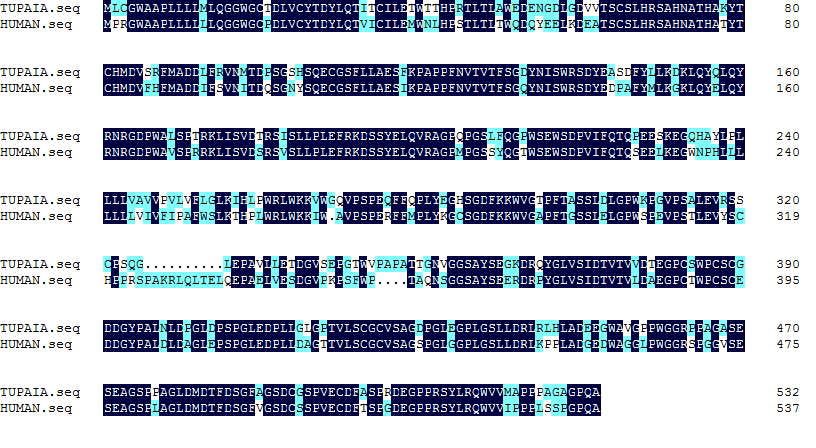

Supplement: S3 Fig — (TIF) [file pone.0176707.s003.tif]

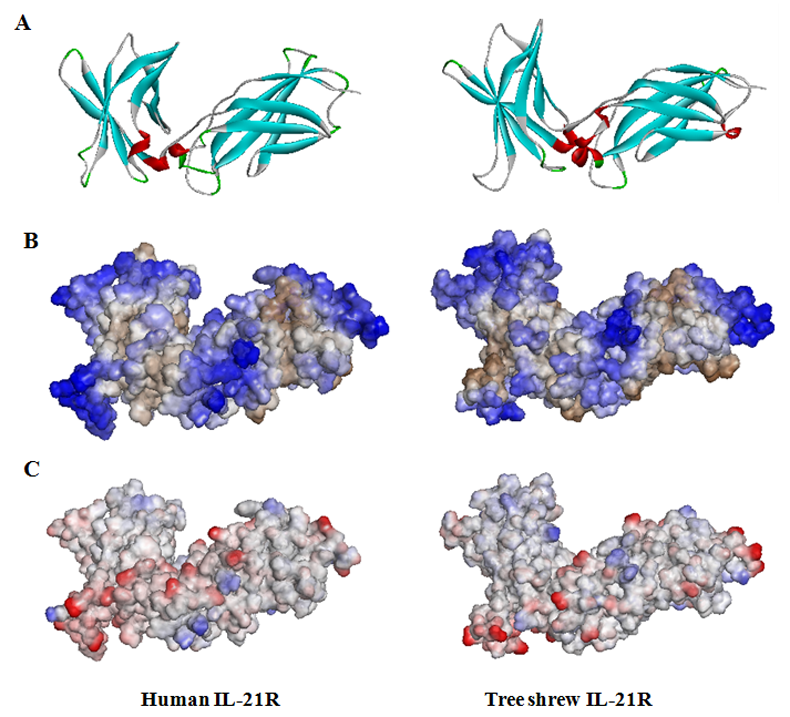

Supplement: S4 Fig — (A) Secondary structure of tree shrew IL-21R (right) compared with human IL-21R (left). Red represents α helices, cyan represents β sheets, green represents β turns, white represents random coils, and yellow represents N-glycosylation sites. (B) Hydrophobicity of tree shrew IL-21R (right) compared with human IL-21R (left). Blue represents hydrophilicity, brown represents hydrophobicity, white represents transition. (C) Surface charge of tree shrew IL-21R (right) compared with human IL-21R (left). Blue represents negative charge, red represents positive charge, white represents no electrical charge. (TIF) [file pone.0176707.s004.tif]
